# CNVind: an open source cloud-based pipeline for rare CNVs detection in whole exome sequencing data based on the depth of coverage

**DOI:** 10.1186/s12859-022-04617-x

**Published:** 2022-03-05

**Authors:** Wiktor Kuśmirek, Robert Nowak

**Affiliations:** Warsaw University of Technology, Institute of Computer Science, Nowowiejska 15/19, 00-665 Warsaw, Poland

**Keywords:** Copy number variation, Read depth, Next-generation sequencing

## Abstract

**Background:**

A typical Copy Number Variations (CNVs) detection process based on the depth of coverage in the Whole Exome Sequencing (WES) data consists of several steps: (I) calculating the depth of coverage in sequencing regions, (II) quality control, (III) normalizing the depth of coverage, (IV) calling CNVs. Previous tools performed one normalization process for each chromosome—all the coverage depths in the sequencing regions from a given chromosome were normalized in a single run.

**Methods:**

Herein, we present the new CNVind tool for calling CNVs, where the normalization process is conducted separately for each of the sequencing regions. The total number of normalizations is equal to the number of sequencing regions in the investigated dataset. For example, when analyzing a dataset composed of *n* sequencing regions, CNVind performs *n* independent depth of coverage normalizations. Before each normalization, the application selects the *k* most correlated sequencing regions with the depth of coverage Pearson’s Correlation as distance metric. Then, the resulting subgroup of $k+1$ sequencing regions is normalized, the results of all *n* independent normalizations are combined; finally, the segmentation and CNV calling process is performed on the resultant dataset.

**Results and conclusions:**

We used WES data from the 1000 Genomes project to evaluate the impact of independent normalization on CNV calling performance and compared the results with state-of-the-art tools: CODEX and exomeCopy. The results proved that independent normalization allows to improve the rare CNVs detection specificity significantly. For example, for the investigated dataset, we reduced the number of FP calls from over 15,000 to around 5000 while maintaining a constant number of TP calls equal to about 150 CNVs. However, independent normalization of each sequencing region is a computationally expensive process, therefore our pipeline is customized and can be easily run in the cloud computing environment, on the computer cluster, or the single CPU server. To our knowledge, the presented application is the first attempt to implement an innovative approach to independent normalization of the depth of WES data coverage.

**Supplementary Information:**

The online version contains supplementary material available at 10.1186/s12859-022-04617-x.

## Background

Copy Number Variation (CNV) has been identified as a major cause of structural variation in the genome, involving both duplications and deletions of sequences [1–3]. Recently, strong rare CNV associations with four major disease categories, including autoimmune, cardio-metabolic, oncologic, and neurological/psychiatric diseases, have been uncovered [4]. Despite the great importance of detecting CNVs, especially rare events, the current CNVs detection tools are characterized by insufficient performance and unsatisfactory classification metrics [5–8].

There are many applications for CNVs detection in Whole Exome Sequencing (WES) data. Many of them use depth of coverage [9]. Applications that use coverage depths typically process data in a few steps: (i) counting coverage in the sequencing regions, (ii) quality control, (iii) normalization, (iv) segmentation and CNVs calling [10].

The most important step in detecting CNVs based on WES depth of coverage is the normalization process, which estimates the ,,perfect” coverage table when there are no CNVs in investigated samples. The goal of obtaining the ,,perfect” coverage table is to eliminate the various sources of biases; the algorithms used for this task are different in other applications. For example, the CODEX [11] and CODEX2 [12] tool adopt a robust iterative maximum-likelihood algorithm based on the WES depth of coverage and exon-wise GC content; the ExomeDepth [13] tool uses the robust beta-binomial logistic model, the CANOES [14] application—the negative binomial distribution, the EXCAVATOR [15] application adopts a median normalization approach for bias removal.

There are also normalization methods that divide the investigated samples into groups. For example, in the [16] paper, we presented that dividing investigated samples based on the correlation between them could improve the resultant set of detected CNVs. What is more, tools like CANOES [14], ExomeDepth [13] and CLAMMS [17] select for each tested sample a set of reference samples (the most correlated) that will be used for background modeling.

The common element of all CNVs detection tools is the normalization process that occurs once for the entire group of sequencing regions, e.g., in the CODEX application for all sequencing regions from a given chromosome all samples set. Herein, we present a completely new approach to the process of normalizing the depth of coverage in sequencing regions. In the presented approach for each sequencing region, the *k* most correlated sequencing regions are chosen, then the resultant set composed of $k+1$ sequencing regions is normalized. Thus normalization is performed for each sequencing region independently; the CNVind application implementing the algorithm is available online https://github.com/wkusmirek/CNVind.

## Implementation

In this section, we present the main data processing pipeline implemented in the CNVind tool. Herein, we presented the workflow of our approach, a detailed description of the processes for calculating the depth of coverage, independent read depth normalization, and CNV calling.

### Workflow

**Fig. 1:**
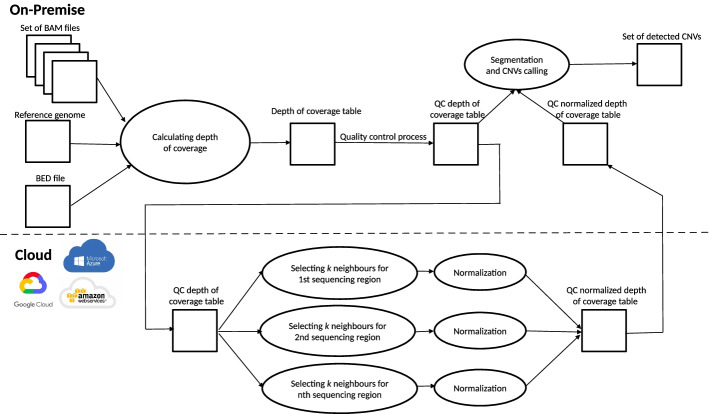
Workflow of the CNVind tool. The first step in data processing in the proposed approach is mapping the DNA reads to the sequencing regions of the reference genome. The mapping result is a matrix of numbers describing the depth of coverage in a given sequencing region. Then, quality control process is applied. After that, for each of the sequencing regions, a set of other sequencing regions is selected to model the background, in our experiments, we examined the selection of the (I) *k* most correlated, and (II) *k* random sequencing regions. As a result of this process, *n* (*n* depicts the number of sequencing regions in the input dataset) subsets of sequencing regions are created, each subset contains $k+1$ sequencing regions. Then, each of the *n* subsets is normalized; from each normalized depth of coverage dataset, the single sequencing region currently under consideration is extracted. Finally, normalized results for individual sequencing regions are combined into a single, normalized matrix; based on the normalized matrix of the coverage depths, raw coverage depths, and coordinates of the sequencing regions, the process of segmentation and CNVs calling is applied. The result of the entire process is the set of detected CNVs. It is worth noting that the normalization process in the proposed approach takes place *n* times for $k+1$ sequencing regions. In contrast, in the CODEX application, the normalization process occurs only once, taking into account the entire set of sequencing regions. Moreover, the process of independently normalizing each sequencing region along with the background modeling subset could be time-consuming so that this step can be performed by the presented CNVind application in a cloud computing environment, on a computer cluster, or a single server.

The workflow of the CNVind tool is presented in Fig. 1. Briefly, the data processing begins by calculating the depth of coverage on each sequencing region. The result of this process is a raw depth of coverage table, where consecutive samples are in columns (number of columns is equal to the number of BAM files specified by the user), in rows—successive sequencing regions (the coordinates of the start and the end of the sequencing region are set by the user at the beginning of the application run). Numerical values of the raw depth of coverage table depict the number of DNA reads mapped in a given sequencing region for specified sample. Then, the quality control process is carried out on the resulting raw depth of coverage table. This process is designed to remove anomalies in samples and sequencing regions. For example, sequencing regions with a very small or very large median depth of coverage, caused by biases of the target capture probes [18], are removed from further analyzes.

Then independent normalization of the depth of coverage in the sequencing region is performed—for each sequencing region, the *k* most correlated other sequencing regions are matched. The normalization module normalizes the resulting subgroups of sequencing regions; the results are combined into one table containing a normalized depth of coverage values. Finally, the resultant set of CNVs is detected based on the raw and normalized depth of coverage tables. The subsequent steps of the data processing listed here are described in detail later in this work.

### Depth of coverage calculations

The first step of CNVs detection is to calculate the depth of coverage in sequencing regions from the input BAM files. There are many applications for calculating the depth of coverage, such as: SeQuiLa-cov [19], samtools depth [20], bedtools genomecov [21], GATK DepthOfCoverage [22], sambamba [23], mosdepth [24]. These applications differ in the degree of parallelization of computations and the approach to counting the DNA reads mapped in a given sequencing region. For example, the ,,pileup” approach is implemented in samtools, GATK and sambamba, while the bedtools, mosdepth and SeQuiLa-cov tools use ,,events” approach. The ,,pileup” approach iterates through each nucleotide at every read in a BAM file while ,,events” approach uses only the start and end positions of the DNA reads. It follows that both methods lead to the same depth of coverage, but the ,,events” approach is much less time-consuming. Despite the mentioned differences, there is no differences in the degree of parallelization, the computation time largely depends on the implementation of the tool, which was compared in the [19] paper. What is more, other tools implement another set of filters to determine whether a given DNA read is counted as correctly mapped or not. Different filters in different tools can significantly affect the numerical values representing the depth of coverage in a given sequencing region.

To overcome this issue, the CNVind application provides a default module for calculating the depth of coverage in sequencing regions. However, this module can be easily replaced—a ready-made raw depth of coverage table can be provided at the input of the CNVind application. This table can be prepared using different applications to calculate the depth of coverage with different values of the parameters filtering the DNA reads. We believe this approach allows for a maximum level of flexibility in terms of calculating the depth of coverage in sequencing regions.

### Read depth normalization

Next, we carried out the process of normalizing the depth of coverage. The proposed approach follows fork-join [25] processing model with each sequencing region being processed separately (possibly in parallel) and combining many outputs into the final normalized depth of coverage table. Operations performed on a single sequencing region include selecting the background modeling set composed of the *k* most correlated sequencing regions, followed by normalization producing a list of normalized depth of coverage values for a considered sequencing region. The union of all partial results creates the final normalized depth of coverage table for the whole input sequencing regions set.

The single normalization process is applied on the set of sequencing regions composed of $k+1$ sequencing regions (*k* depicts the number of background modeling sequencing regions, $+1$ results from adding single investigated sequencing region to the background modeling set). In the CNVind tool for normalization, we used the normalization module implemented in the CODEX tool [11]. Briefly, the approach uses a Poisson log-linear model including terms that specifically remove biases due to exon length, GC content, capture, amplification efficiency, and latent systematic artifacts. The most likely model parameters are estimated based on maximum likelihood estimation (MLE) [26, 27].

The aspect that distinguishes CNVind applications from other state-of-the-art applications is the approach to the normalization process. Previous tools have only performed one normalization on the entire set of sequencing regions available, e.g. on all sequencing regions from a given chromosome. The presented approach is innovative, for each sequencing region a set of *k* most strongly correlated other sequencing regions is selected, the resulting subgroups are normalized, the results are combined into the resulting normalized depth of coverage table. The k-nearest neighbors [28] algorithm with the depth of coverage Pearson’s Correlation as distance metric is used to determine *k* strongly correlated sequencing regions.

### CNVs calling

After the depth of coverage normalization step, a CNVs calling is performed. The process is iteratively performed for subsequent samples based on the raw and normalized depth of coverage values. If the normalized number of reads (the predicted number of reads in the absence of CNV) is much greater than the raw number of mapped reads, then deletion is likely to be present in that sequencing region. On the other hand, if the value of the normalized depth of coverage is significantly smaller than the raw value of the coverage depth, then there is a probable duplication in the given sequencing region. Thus, we have a ratio of raw coverage depth to normalized coverage depth for each sequencing region in a given sample. For ratios greater than 1, there is a duplication probability, for coefficients less than 1—a deletion probability. Finally, based on the mentioned ratios and the distances between sequencing regions, some of them are merged to avoid a large number of very small CNVs. For this purpose, the CNVind application implements the circular binary segmentation algorithm [29].

### Application architecture

The CNVind tool consists of four independent modules responsible for: (I) calculating depth of coverage, (II) removing some sequencing regions and samples in quality control process, (III) selecting *k* mostly correlated sequencing regions and normalization, (IV) segmentation and CNVs calling. Each of the mentioned modules is built and deployed as a separate docker [30] image that can be run both on a local computer, on a computer cluster or in a cloud computing environment. In order to speed up the calculations during the step of independent selection of the *k* most correlated sequencing regions and normalization of the depth of coverage, the calculations can be parallelized using the Kubernetes [31] container orchestration tool. Kubernetes (K8s) is an open-source system for management, scaling and deployment of containerized applications. In the CNVind tool Kubernetes, for each sequencing region, calls a docker instance which processes and normalizes the data for a given sequencing region. After the calculations are finished and the results are saved, Kubernetes releases the resource, appointing a new docker for the next sequencing region. Depending on the available resources, Kubernetes can run several dockers at the same time allowing for almost linear time scalability. An additional advantage of using Kubernetes is portability—it can be installed and run on Amazon Web Services (AWS), Microsoft Azure, the Google Cloud Platform (GCP), or in on-premise installations.

At this point it should be mentioned that the most time-consuming stage of data processing in the CNVind application is the normalization of the depth and coverage. The input of this process is composed of two tables: (I) table with the raw depth of coverage values, (II) table with description of sequencing region coordinates. The sizes of both tables are not large, table (I) in our study for the entire chromosome 1 was 54 MB, and 29 MB for chromosome 11, table (II) is the size of a 7 MB. The normalization process is independent between regions, each region can be processed in parallel by a different docker/pod. After completing the docker/pod calculations, it creates a result file with normalized depth and coverage values for a given sequencing region. The input files are accessed through a shared directory—when running calculations on a computer cluster, depending on the cluster, the input files should be placed in a directory that is visible from all nodes—for example, for Kubernetes on the Microsoft Azure computing cloud, Storage Class structure and Persistent Volume Claim should be created, and then the input files should be placed in the appropriate directory. This way, each pod run under Persistent Volume Claim can access input files from Storage Class, there is only a single instance of the input file for all running pods. Similarly, after the computations are completed for the normalization results, each pod creates a result file with normalized values of the coverage depth for a given sequencing region. The results file is saved to the same Storage Class. When all pods finish their calculations, the resulting files are merged into a single file with a normalized coverage table. The duration of access to the input data and saving the results is negligibly small.

## Results

This section describes the experiment results and CNVs detected by the CNVind tool presented in the paper. To evaluate the results, we compared the detected set of CNVs with the CNVs set golden record provided by 1000 Genomes Consortium [32] generated based on the Whole Genome Sequencing (WGS) data. In conducted evaluation process all CNVs sets were categorized based on: (I) frequency: rare (frequency$\le$1%), common (frequency>1%) CNVs and (II) length: short (encompassing 1 or 2 exons) and long (encompassing more than 2 exons) CNVs. Moreover, in the diagrams presenting the results of the evaluation process there is also the group ,,all”, which means all CNVs, that is, for the length filter: ,,all” = ,,short” + ,,long”, and for the frequency division: ,,all” = ,,rare” + ,,common”. We compared the performance of the presented CNVind tool with state-of-the-art CNVs detection applications: CODEX ver. 1.22 and exomeCopy ver. 1.36.

### Benchmark dataset

We tested the performance of the CNVind tool on a public dataset consisting of 861 samples from 1000 Genomes Project [32]. The investigated sample set consisted of 444 females and 417 males, including 205 samples from Europe, 276 samples from Africa, 313 samples from Asia, and 67 samples from America. To reduce the computation time, we considered chromosome 11 only. However, we repeated the entire analysis for chromosome 1 to assess the potential impact of chromosomal variability on the final results (the results presented in the Additional file 1).

Before the experiments, we carried out the quality control process to remove sequencing regions that did not pass the quality filters. In this process, we removed all sequencing regions (I) with GC content below 20% or greater than 80%, (II) with mappability factor below 0.9, (III) with median read depth across all samples below 20 or greater than 4000, and (IV) shorter than 20 bp or longer than 2000 bp. All previously mentioned filters and threshold values were transferred from the standard quality control process implemented in the CODEX tool. As a result of the quality control process, 2273 out of 20106 sequencing regions for chromosome 1 and 966 out of 10565 sequencing regions from chromosome 11 were removed.

### Correlation between sequencing regions

**Fig. 2:**
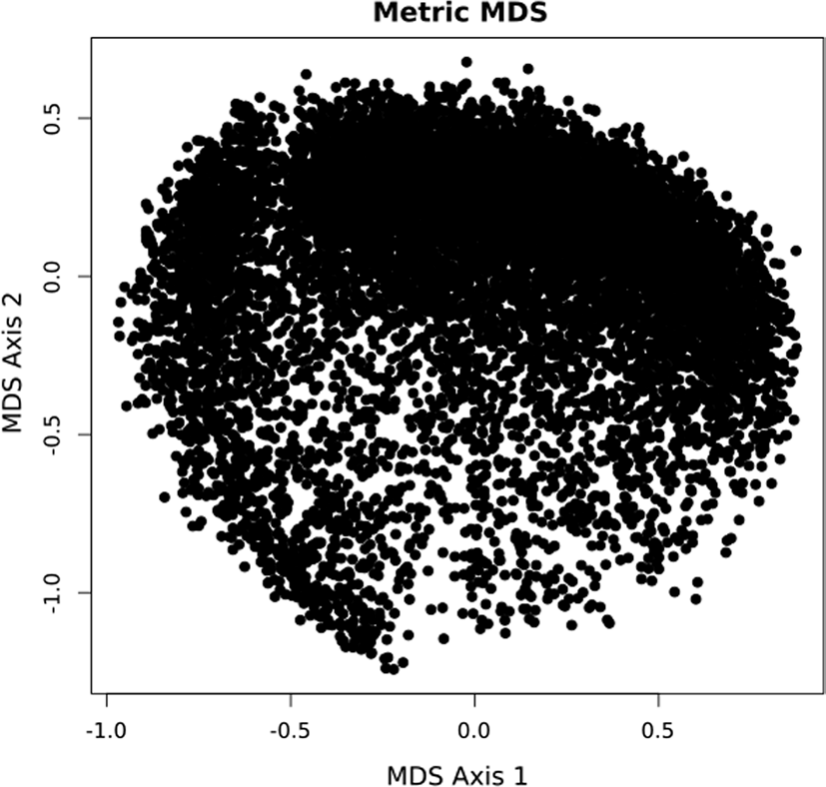
Correlation between depth of coverage in sequencing regions of benchmark dataset (chromosome 11). The figure presents the results of a multidimensional scaling [36] of the covariance matrix of the read count data for the 9599 investigated sequencing regions onto a two-dimensional plane. Each of the dots in the figure represents a sequencing region, the distance between the dots represents the correlation between the depth of coverage of the given sequencing regions. It is worth noticing that there is no clear division into groups; all sequencing regions constitute a relatively uniform and compact group of points. The figure was prepared by R’s cmdscale function.

After the quality control process, we calculated the correlation between sequencing regions based on the depth of coverage across all investigated samples. The results proved that the correlation between the sequencing regions is not uniform—for each sequencing region, different sequencing regions can be designated either very strongly or very weakly correlated. The graphical representation of the depth of coverage across all investigated samples correlation between the different sequencing regions is presented in Fig. 2.

### Performance evaluation

**Fig. 3:**
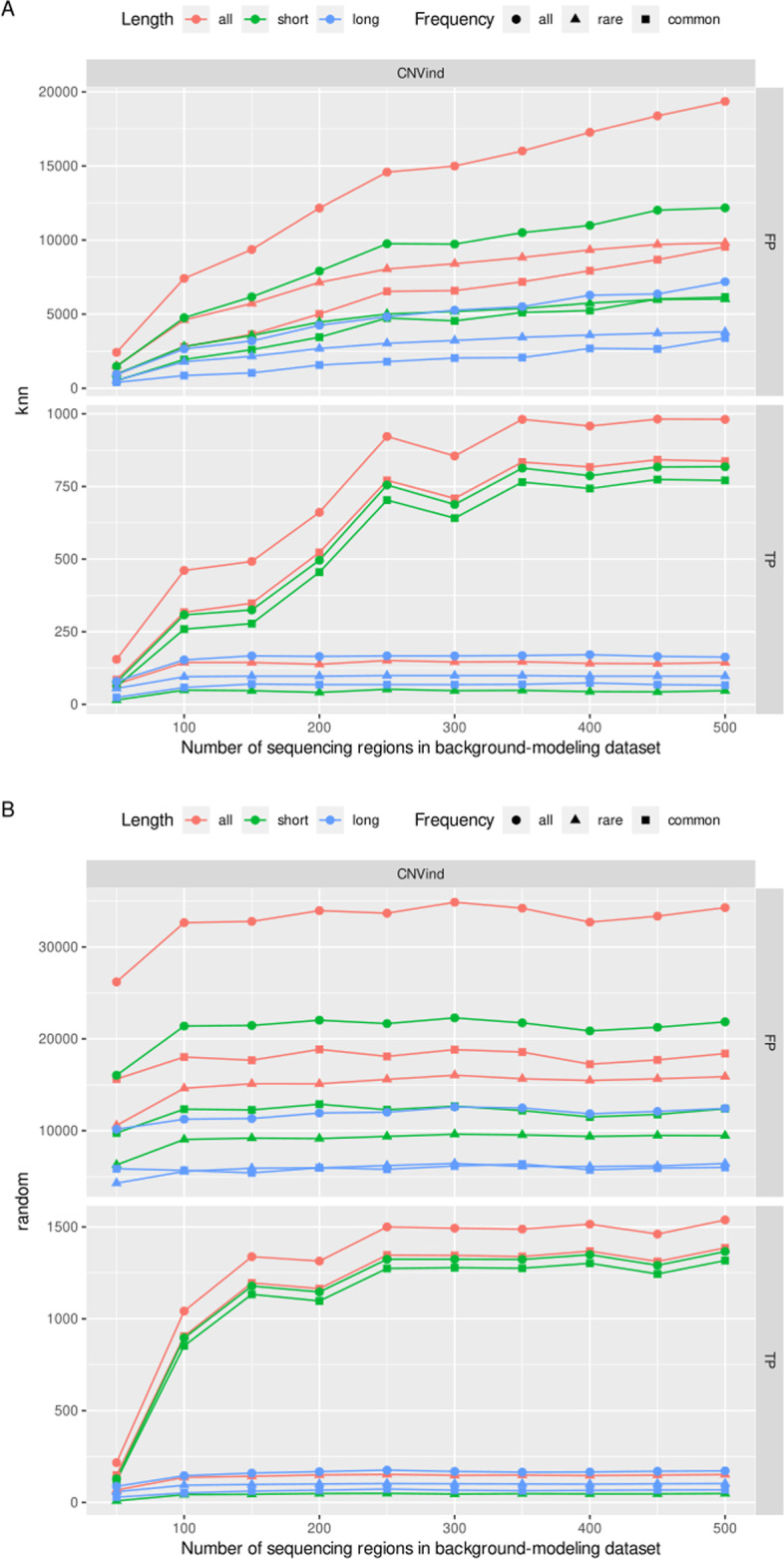
Effect of the size of the set of sequencing regions which models background on the number of CNVs detected by the CNVind tool. It is worth paying attention to the characteristics of rare CNVs in the knn algorithm. As the background modeling set is reduced (to a value equal to 100), the FP number drops drastically while the TP number remains stable. What is more, in both methods of selecting the sequencing regions (knn and random) that model the background, the small size of the background-modeling dataset leads to a decrease in the number of TP and FP calls in all CNVs subgroups.

Firstly, we examined how the number of neighbors in the knn algorithm affects the CNVs detection process results. The obtained results (Fig. 3a) present a very positive impact of the independent normalization process on the resulting set of rare CNVs detected—as the number of sequencing regions in the background-modeling dataset decreases, the number of FP calls decreases. In contrast, the number of TP calls remains constant. The only exception is a tiny number of sequencing regions in the background-modeling dataset (less than 100), then for rare CNVs, both the number of FP calls and TP calls decreases. For common CNVs, the number of detected CNVs—both TP and FP—decreases as the number of sequencing regions in the background-modeling dataset decreases. To sum up this part of results, the value of *k* should not be too small (with too low a value of *k*, the group of normalized sequencing regions is small, so the normalization uncertainty is large) or too large (with a large value of the parameter *k*, the group of normalized sequencing regions is large, the normalization itself gives an exact results).

Secondly, we compared the knn algorithm with the random selection of sequencing regions that model the background, and the results are presented in Fig. 3b. For the random method, the number of detected CNVs that are TP and FP is constant, regardless of the number of background-modeling sequencing regions. The only exception to this rule is the small set of background-modeling sequencing regions—here, both TP and FP numbers decrease as the background-modeling set decreases in size.

**Fig. 4:**
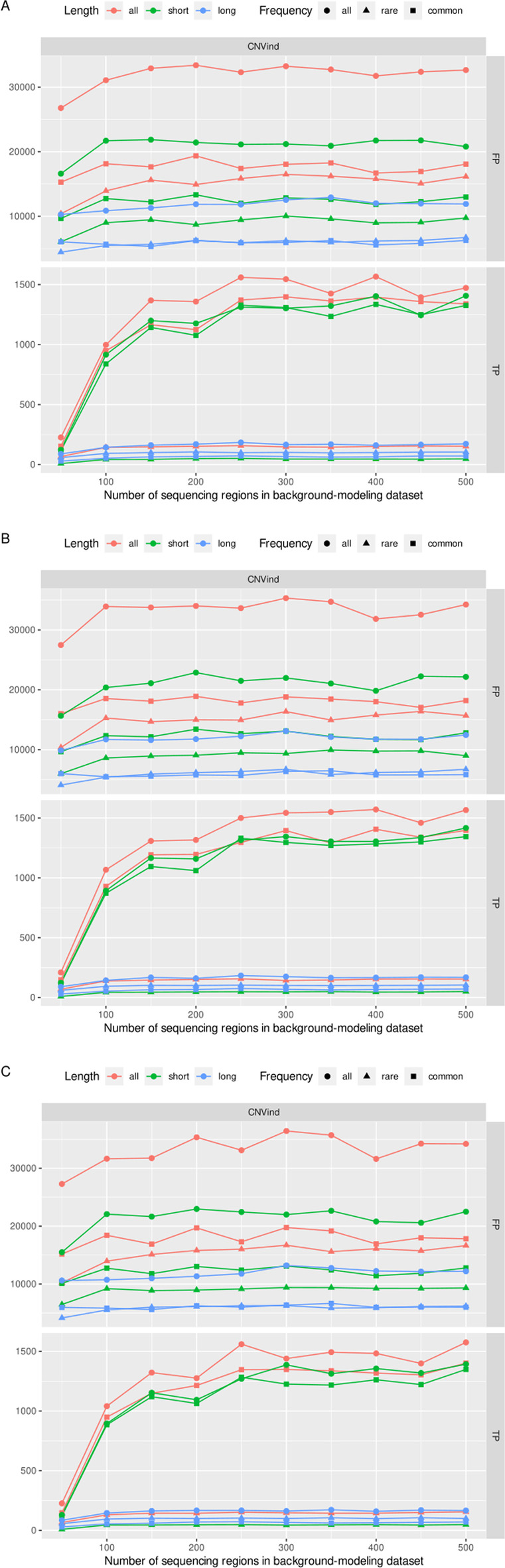
Results for knn algorithm with another metrics of similarity between sequencing regions. In the diagram, we presented the results for other metrics of similarity between sequencing regions: **a** GC content, **b** the length of the sequencing region, and **c** the mean depth of coverage in the sequencing regions. The obtained results are very similar to the random selection of the *k* most similar background modeling sequencing regions

Thirdly, we checked whether other similarity metrics between the sequencing regions would yield better results. For this purpose, we tested three measures of similarity between the sequencing regions: (I) GC content, (II) the length of the sequencing region, and (III) the mean depth of coverage in the sequencing region. The obtained results are presented in Fig. 4. The results obtained presented that taking the three mentioned metrics to measure the similarity between the sequencing regions did not improve the results. Moreover, the obtained results are very similar to the random selection of the *k* most similar background modeling sequencing regions. This fact concludes that the depth of coverage Pearson’s Correlation as distance metric is the best metric to determine *k* strongly correlated sequencing regions.

**Fig. 5:**
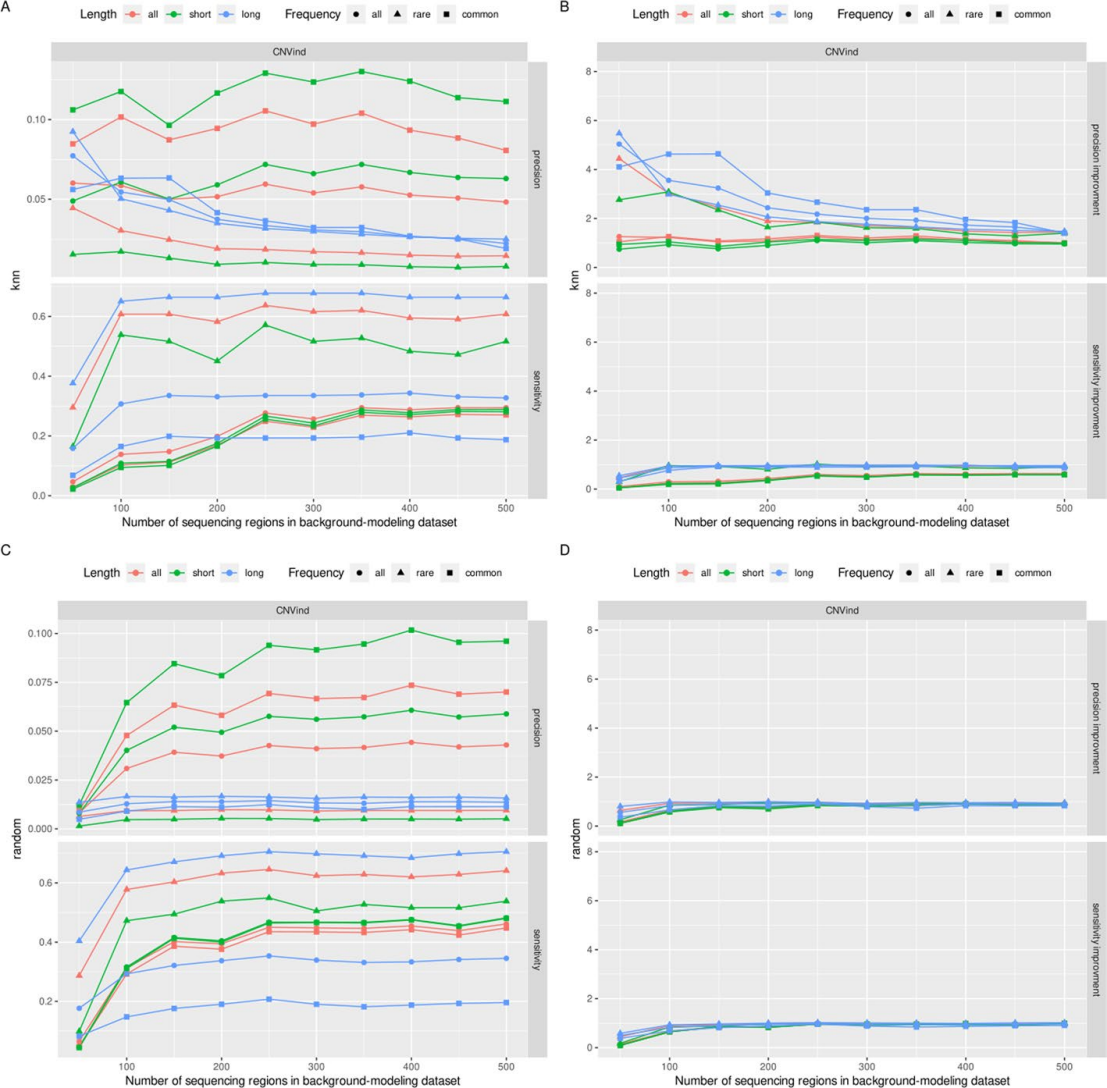
Effect of the size of the set of sequencing regions which models background on the results’ sensitivity and precision. **a, b** relate to the selection of the k most correlated sequencing regions, **c, d** k random sequencing regions. Additionally, **b, d** the improvement of individual results relative to the baseline, i.e. all sequencing regions are normalized simultaneously. It is worth paying attention to the fact that reducing the value of *k* in the knn algorithm to a value equal to 100 (**b**) allowed for a 3-fold improvement in precision while maintaining a constant level of sensitivity for rare CNVs

Lastly, we examined how the number of neighbors in the knn and random algorithm affects the results’ precision and sensitivity; the results are presented in Fig. 5. The diagram proves that as the value of *k* decreases in the knn algorithm for rare CNVs, the precision increases, while the sensitivity remains constant. Only for a very small value of *k* (below 100) the sensitivity for rare CNVs also decreases. The diagram also shows that changing the value of *k* for the random algorithm does not significantly affect the sensitivity and precision of rare CNVs detection process. The only exception is a very small *k* value where the detection sensitivity of rare CNVs decreases.

**Fig. 6:**
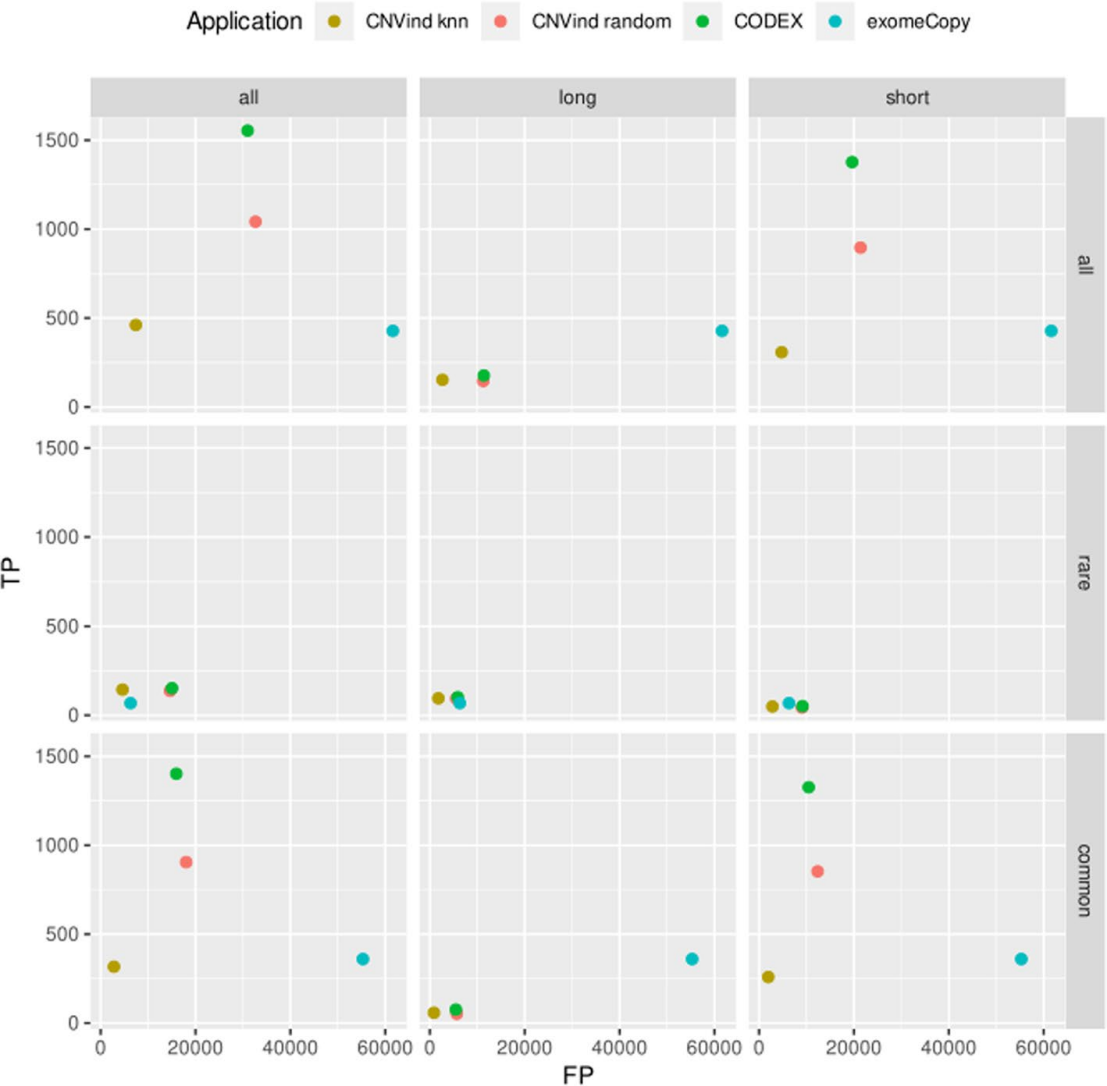
Comparison of the results obtained by the CODEX, exomeCopy, and CNVind applications (in knn and random modes). The diagram presents a comparison of the CODEX, exomeCopy, and CNVind tools. We ran the CNVind tool in two modes: (I) knn (independent normalization of each sequencing region with the other *k* most correlated sequencing regions), (II) random (independent normalization of each sequencing region with the other *k* random sequencing regions). In the diagram, the *k* value for the knn and random algorithms is equal to 100. It is worth noting that for rare CNVs (middle row of the diagram), the CNVind application with knn algorithm is the best-evaluated tool for detecting CNV events, and the results obtained from the CODEX tool and the CNVind application with random algorithm are almost identical—the dots on the diagram nearly match. Moreover, the normalization module from the CODEX application has been implemented in the CNVind application; therefore, the CNVind application with the *k* parameter equal to the number of sequencing regions in the chromosome (minus the currently considered sequencing region) gives the same set of CNVs. Thus, by comparing the results obtained by the CNVind and CODEX applications, we can see how the independent normalization described in the presented paper positively influences the resulting set of detected CNVs

What is more, we compared the two previously presented methods of selecting background-modeling sequencing regions (knn and random) with the methods implemented in the CODEX and exomeCopy tools. There is only single normalization for all sequencing regions from a given chromosome. The results of our experiment are presented in Fig. 6. The diagram presents that the results for the CODEX and CNVind with the random method for rare CNVs are almost identical (middle row of the graphs). However, the CNVind with knn approach for rare calls significantly reduces FP events compared to the CODEX method keeping a stable number of TP calls. What is more, the CNVind application allows for better results also than the exomeCopy tool.

### Computation time

**Fig. 7:**
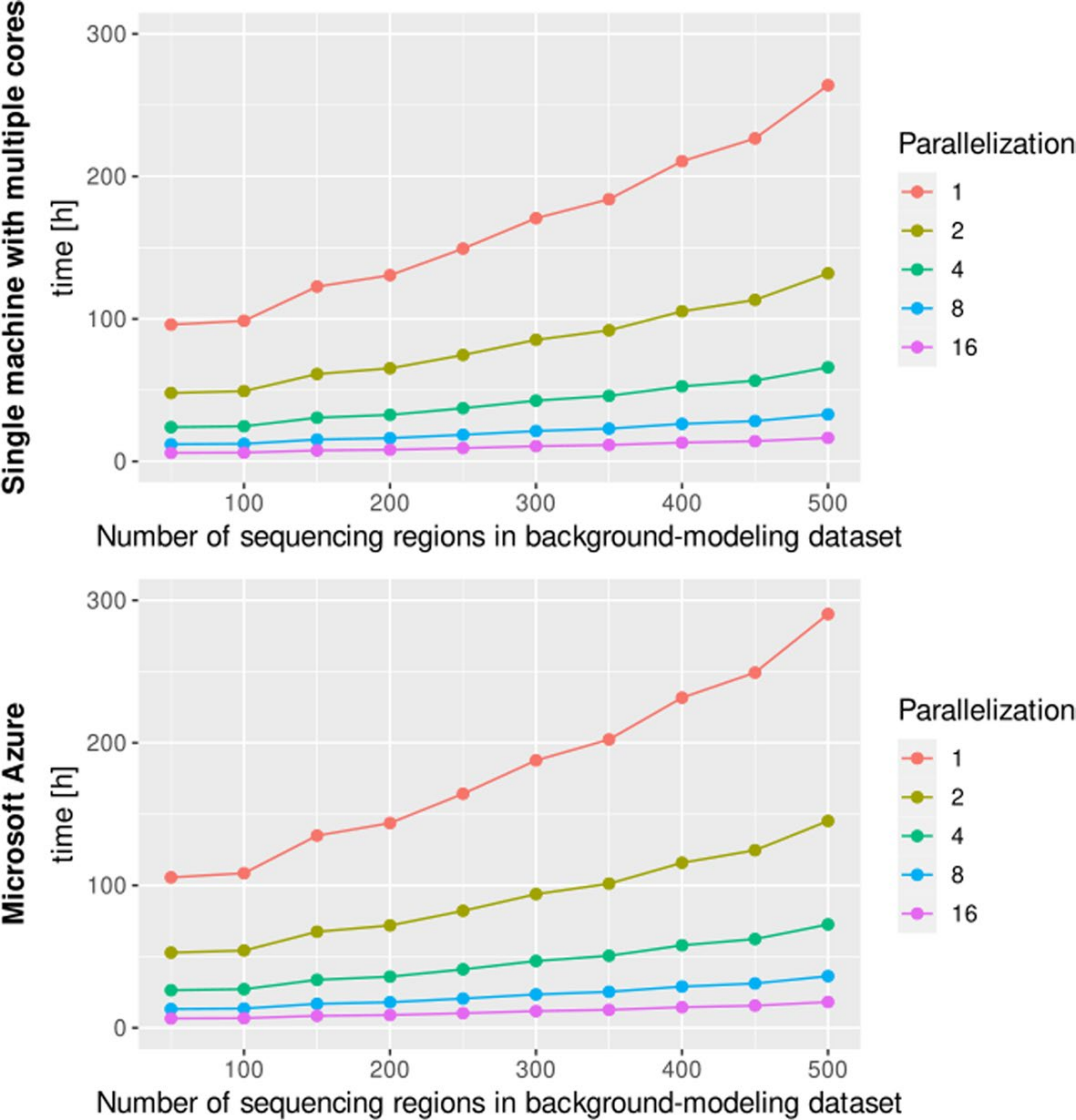
Comparison of the depth of coverage normalization computation times for the CNVind application on a single machine with multiple cores and in Microsoft Azure cloud. The diagram presents the changes in the computation time for the CNVind tool in the Microsoft Azure cloud and on a single machine with multiple cores. The comparison showed that as the degree of parallelization increases, the computation time decreases linearly. As the degree of parallelization of calculations in the case of (I) the single machine with multiple cores, we assume the number of Docker images working in parallel, and (II) in the case of the Microsoft Azure cloud—the number of pods working in parallel. For comparison, the computation time for the baseline version of the CODEX and exomeCopy tool (only a single normalization process occurs for all sequencing regions) is equal to 14 min 38 s and 12 min 52 s, respectively.

Finally, we examined and compared the computation time of the presented approach; the results of the experiment are presented in Fig. 7. The presented approach is significantly slower than the implementation of the CODEX and exomeCopy—in our approach, normalization occurs independently for each sequencing region. In the original implementation of the CODEX and exomeCopy application, normalization is performed only once for all sequencing regions from a given chromosome. However, the process of independent normalization can be easily parallelized, and the presented pipeline is adapted to be used in a parallel manner in the cloud computing environment or on the computer cluster.

## Discussion

In this paper, we presented the CNVind tool, the new application for calling CNVs based on the depth of coverage in WES data. The main innovation of the presented approach is the parallelized process of the independent depth of coverage normalization of each sequencing region. This stage is time-consuming, but the proposed pipeline is implemented in such a way that the application can be run on a computer cluster or in a cloud computing environment—the degree of parallelization depends on the available computing resources. In particular, the application can be run in the Kubernetes system, each pod normalizes the depth of coverage in a different sequencing region, only single instance of input files is stored on the cluster in a properly created Storage Class object with access via the Persistent Volume Claim structure. The same Storage Class represents the place where the results of the pods computations are stored.

The main advantage of the presented application is the ability to improve the detection results of rare CNVs. In the experiments presented in the study, we showed that the use of the CNVind application allowed for a three-fold reduction in the number of rare FPs in relation to the results obtained from the original CODEX application. What is more, the significant reduction in the number of FPs did not reduce the number of rare TPs detected. Moreover, the paper presents the results for chromosome 11, additional results of the same experiments on chromosome 1 presented in Additional file 1 showed that this regularity is also maintained for another set of input data.

There are two main disadvantages of the proposed approach. Firstly, the normalization of the sequencing regions is independent, which results in a longer computation time than one normalization of all sequencing regions simultaneously. This disadvantage is solved by the maximum dispersion of computations and adapting the application to computing in the cloud computing environment. Secondly, for the application to function properly, there must be a sufficiently large number of sequencing regions that are currently explored. In the presented application, the *k* most correlated sequencing regions are selected for each sequencing region, which models the background. Therefore, it is important that the set of sequencing regions from which the final background modeling subset is selected should be large enough. For example, in this article, we presented the results for the analysis of WES data from chromosome 1 and chromosome 11, which contained the 20106 and 10565 regions of sequencing, respectively.

One of the main directions of the application development is the addition of a module that would automatically select the *k* parameter, i.e., the number of the most correlated sequencing regions used for background modeling during independent normalization. In the article, we present the research results for *k* assuming values in the range 50 to 500. However, the optimal value of *k* depends on the investigated data set. The planned approach to the effective determination of the value of *k* will be based on the approach presented in the Ximmer [33] tool. The mentioned application simulates artificial CNVs and implants them into the input data set. Then, it selects the parameters for triggering the application so that the sensitivity and specificity of the detection of artificial, simulated CNVs are as high as possible. In our approach, we plan to do the same, optimizing the value of the *k* parameter; we plan to use the algorithm implemented in the SECNVs [34] and Bamgineer [35] tools for artificial CNVs simulations.

## Conclusions

The presence of rare CNVs causes many genetic diseases. However, detection methods for rare CNVs based on the depth of coverage of WES data are still unsatisfactory, mainly due to the presence of a very high number of false positives calls in the resulting CNVs dataset. Herein, we presented the new CNVind tool with the independent method of normalizing the depth of coverage in sequencing regions, which significantly improves the resulting set of detected CNVs, especially rare CNVs.

## Availability and requirements

Project name: CNVind

Project home page: https://github.com/wkusmirek/CNVind

Operating system(s): All (cross-platform)

Programming language: bash, R

Other requirements: docker

License: GNU GPL-3.0

Any restrictions to use by non-academics: none

## Supplementary Information

**Additional file 1**. Additional results of thesame experiments on chromosome 1. Fig. S1. Correlation between depthof coverage in sequencing regions of benchmark dataset; Fig. S2.Effect of the size of the set of sequencing regions which modelsbackground on the number of CNVs detected by the CNVind tool; Fig.S3. Effect of using other metrics when selecting the k most similarsequencing regions: (A) GC content, (B) sequencing region length,(C) mean depth of coverage in the sequencing region; Fig. S4. Effectof the size of the set of sequencing regions which models backgroundon the results' sensitivity and precision; Fig. S5. Comparison ofthe results obtained by the CODEX, exomeCopy, and CNVindapplications in knn and random modes; Fig. S6. Comparison of thedepth of coverage normalization computation times for the CNVindapplication on a single machine with multiple cores and in MicrosoftAzure cloud.

## Data Availability

Supplementary information, benchmarking procedure as well as test data are publicly accessible at the GitHub repository: https://github.com/wkusmirek/CNVind
