# Supplementary material for: CNVind: an open source cloud-based pipeline for rare CNVs detection in whole exome sequencing data based on the depth of coverage
Source: BMC Bioinformatics. 2022 Mar 5;23:85. doi: 10.1186/s12859-022-04617-x (PMC8897915; doi:10.1186/s12859-022-04617-x)
Supplement: Supplementary file 1 — Additional file 1. Additional results of thesame experiments on chromosome 1. Fig. S1. Correlation between depthof coverage in sequencing regions of benchmark dataset; Fig. S2.Effect of the size of the set of sequencing regions which modelsbackground on the number of CNVs detected by the CNVind tool; Fig.S3. Effect of using other metrics when selecting the k most similarsequencing regions: (A) GC content, (B) sequencing region length,(C) mean depth of coverage in the sequencing region; Fig. S4. Effectof the size of the set of sequencing regions which models backgroundon the results' sensitivity and precision; Fig. S5. Comparison ofthe results obtained by the CODEX, exomeCopy, and CNVindapplications in knn and random modes; Fig. S6. Comparison of thedepth of coverage normalization computation times for the CNVindapplication on a single machine with multiple cores and in MicrosoftAzure cloud. [file 12859_2022_4617_MOESM1_ESM.pdf]

CNVind - An open source cloud-based pipeline for rare CNVs detection in whole exome sequencing data based on the depth of coverage

Wiktor Kuśmirek, Robert Nowak

Supplementary Materials

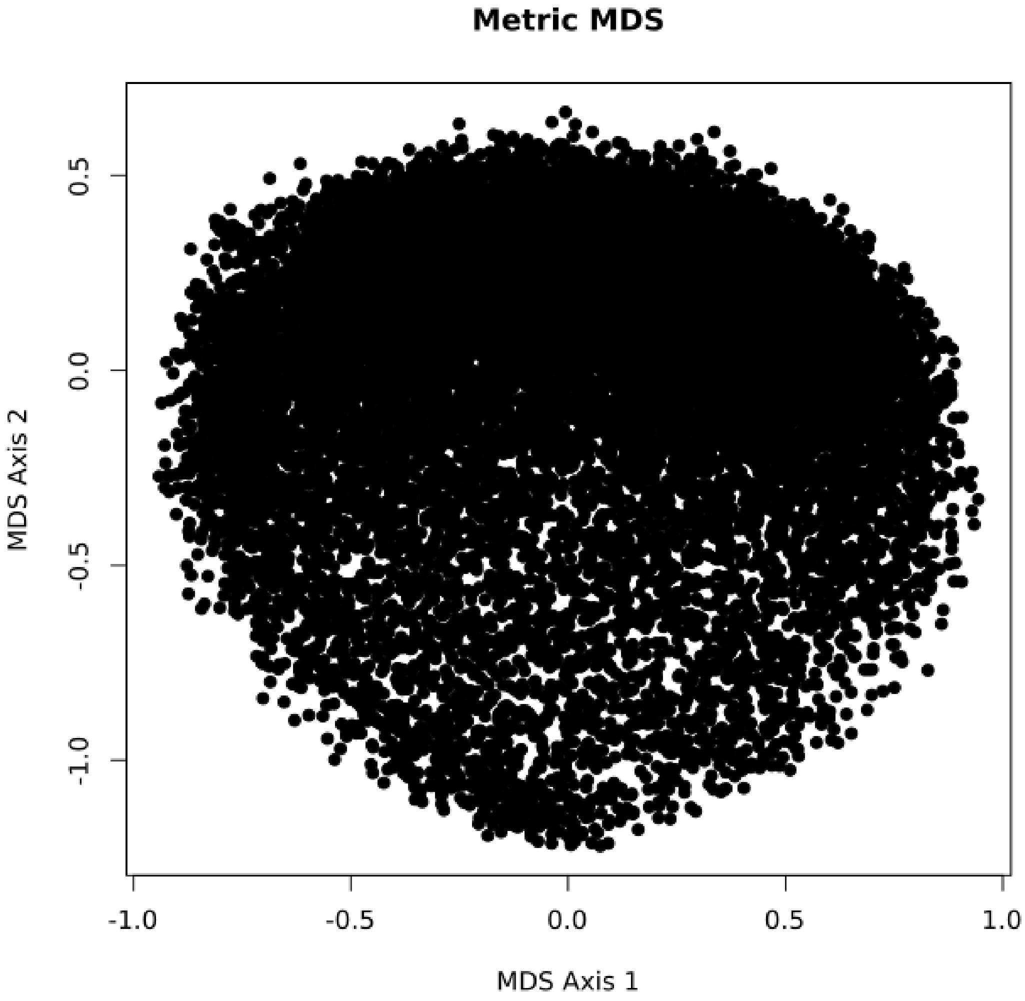

Figure S1: **Correlation between depth of coverage in sequencing regions of benchmark dataset (chromosome 1).**

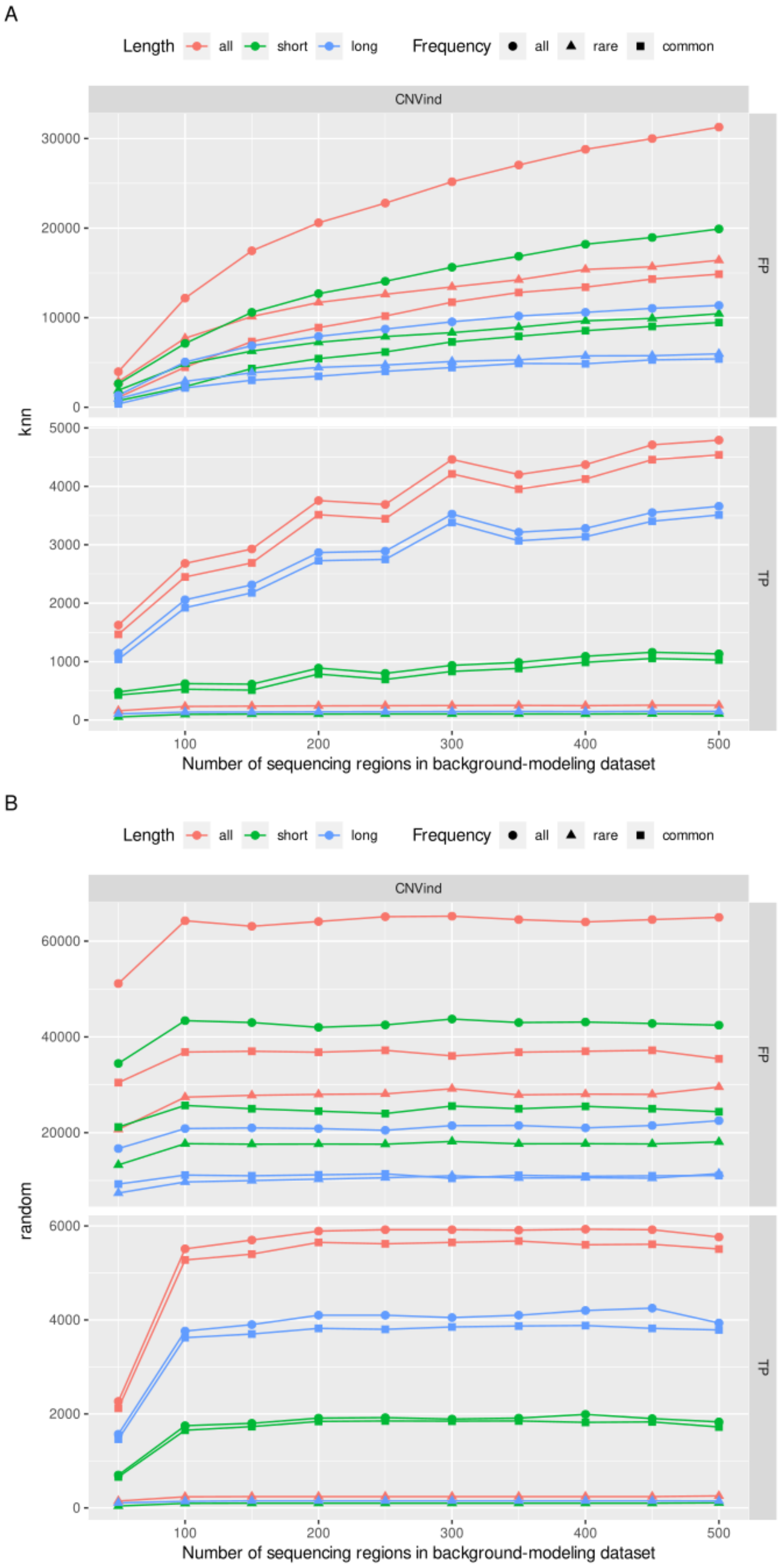

Figure S2: **Effect of the size of the set of sequencing regions which models background on the number of CNVs detected by the CNVind tool (chromosome 1).**

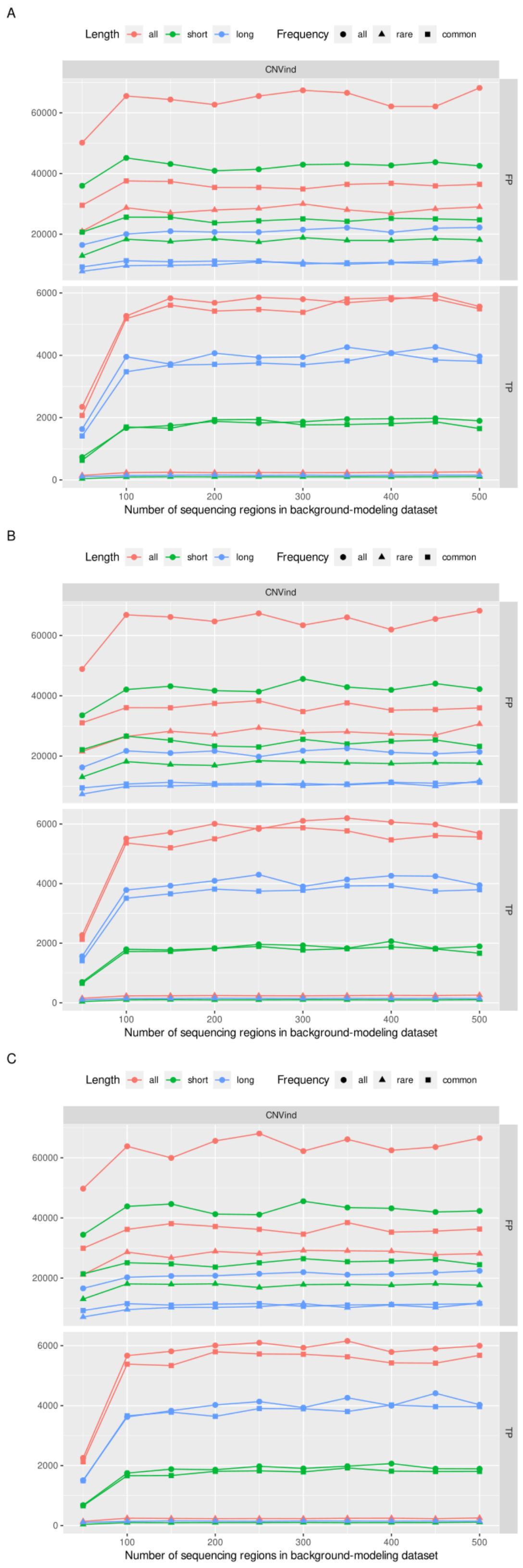

Figure S3: **Effect of using other metrics when selecting the k most similar sequencing regions: (A) GC content, (B) sequencing region length, (C) mean depth of coverage in the sequencing region (chromosome 1).**

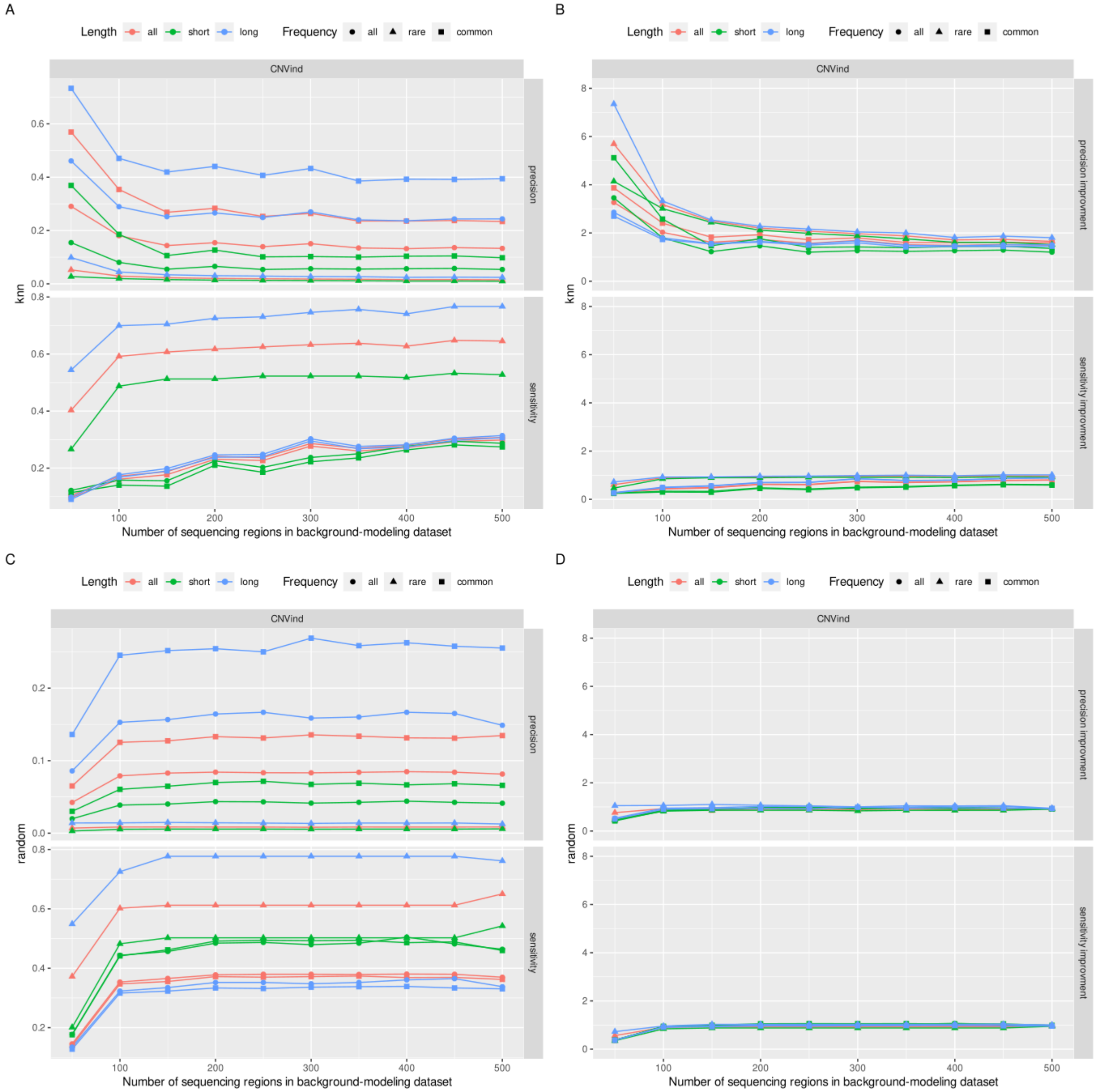

Figure S4: **Effect of the size of the set of sequencing regions which models background on the results' sensitivity and precision. (chromosome 1).**

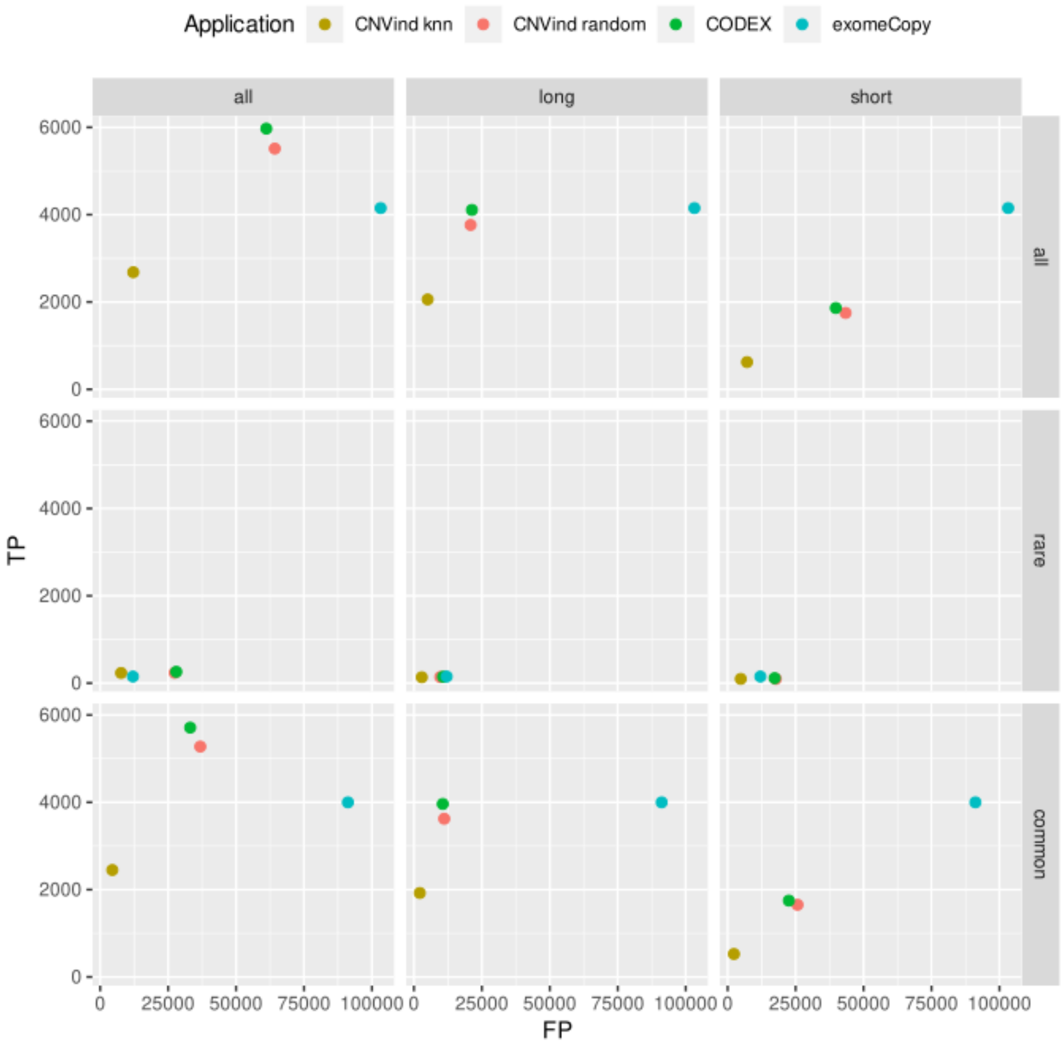

Figure S5: **Comparison of the results obtained by the CODEX, exomeCopy, and CN-Vind applications in knn and random modes (chromosome 1).**

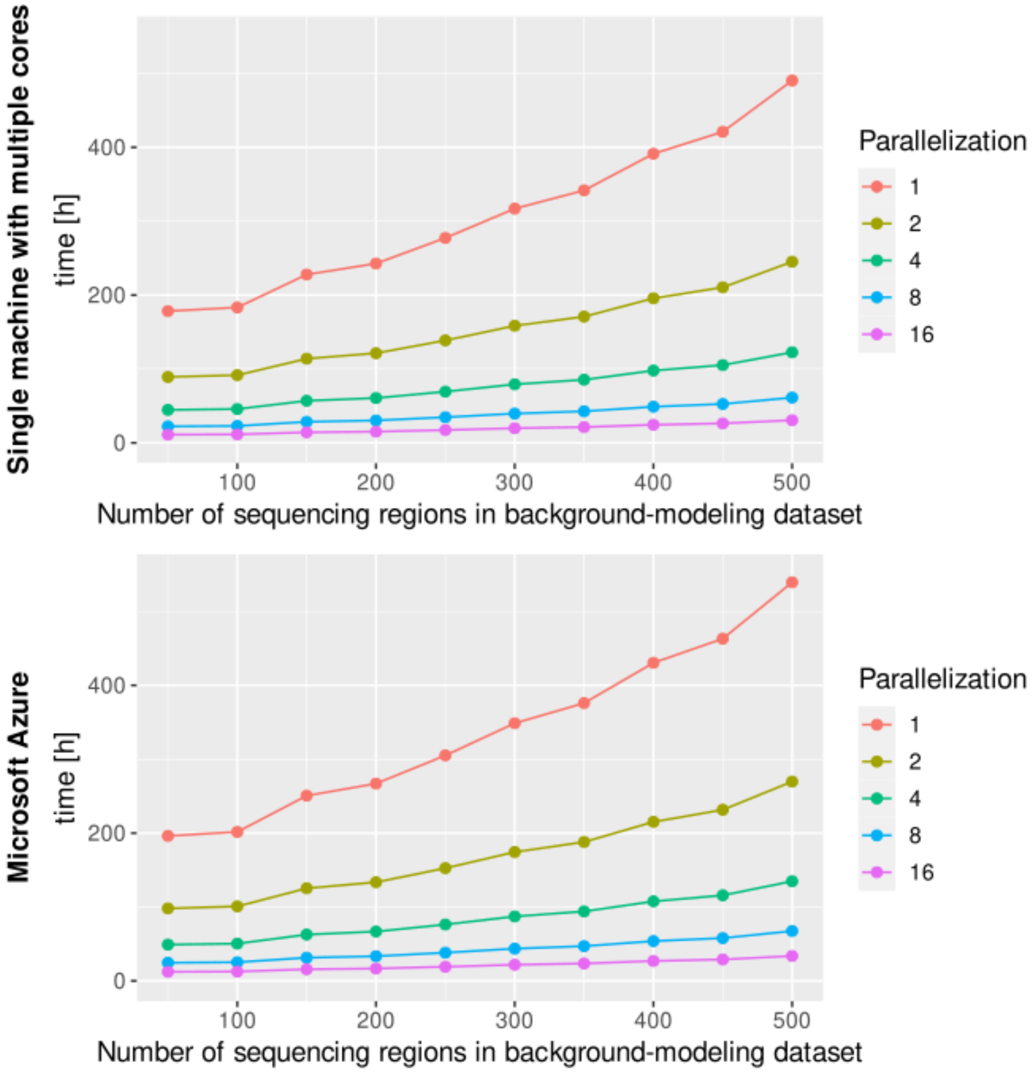

Figure S6: **Comparison of the depth of coverage normalization computation times for the CNVind application on a single machine with multiple cores and in Microsoft Azure cloud (chromosome 1).**
